# Untargeted metabolomics approach for unraveling robust biomarkers of nutritional status in fasted gilthead sea bream (*Sparus aurata*)

**DOI:** 10.7717/peerj.2920

**Published:** 2017-01-26

**Authors:** Ruben Gil-Solsona, Jaime Nácher-Mestre, Leticia Lacalle-Bergeron, Juan Vicente Sancho, Josep Alvar Calduch-Giner, Félix Hernández, Jaume Pérez-Sánchez

**Affiliations:** 1 Research Institute for Pesticides and Water (IUPA), University Jaume I, Castellón, Spain; 2 Institute of Aquaculture Torre de la Sal (IATS, CSIC), Ribera de Cabanes, Castellón, Spain

**Keywords:** Aquaculture, Nutrition, Chromatography, Mass spectrometry, Gilthead sea bream, Serum metabolomics

## Abstract

A metabolomic study has been performed to identify sensitive and robust biomarkers of malnutrition in farmed fish, using gilthead sea bream (*Sparus aurata*) as a model. The metabolomic fingerprinting of serum from fasted fish was assessed by means of ultra-high performance liquid chromatography coupled to quadrupole time-of-flight mass spectrometry. More than 15,000 different *m/z* ions were detected and Partial Least Squares–Discriminant analysis allowed a clear differentiation between the two experimental groups (fed and 10-day fasted fish) with more than 90% of total variance explained by the two first components. The most significant metabolites (up to 45) were elucidated on the basis of their tandem mass spectra with a broad representation of amino acids, oligopeptides, urea cycle metabolites, L-carnitine-related metabolites, glutathione-related metabolites, fatty acids, lysophosphatidic acids, phosphatidylcholines as well as biotin- and noradrenaline-related metabolites. This untargeted approach highlighted important adaptive responses in energy and oxidative metabolism, contributing to identify robust and nutritionally-regulated biomarkers of health and metabolic condition that will serve to assess the welfare status of farmed fish.

## Introduction

Fish aquaculture is the sector of animal livestock production with higher growth rates at the global level. This industry highly contributes to cover the current but also the future demand of nutritious quality food for human consumption (Ottinger, Clauss & Kuenzer, 2016). This starts with the selection of high quality raw materials in order to ensure the development of an efficient and environmentally sustainable sector. However, we need to refine our knowledge on nutrient requirements to produce more robust, safe and quality fish, especially with the advent of new diet formulations based on alternative plant ingredients rather than marine feedstuffs (Karalazos et al., 2007; Médale et al., 2013; Benedito-Palos et al., 2016). As a result, research in fish nutrition is moving from classical methodologies to omics approaches, including transcriptomics (Benedito-Palos, Ballester-Lozano & Pérez-Sánchez, 2014; Louro, Power & Canario, 2014), proteomics (Rodrigues et al., 2012; Wrzesinski et al., 2013) and metabolomics (Kullgren et al., 2010; Silva et al., 2014; Asakura et al., 2014).

Unlike nucleic acid or protein-based omic techniques, metabolomics has to deal with low-molecular weight metabolic entities (<1,000 Da) with diverse chemical and physical properties (Kell, 2004), which can vary from millimolar to picomolar concentrations. Two are the main analytical platforms currently used in metabolomics studies: nuclear magnetic resonance (NMR) (Emwas, 2015) and mass spectrometry (MS) (Castro-Puyana & Herrero, 2013). Most of the studies of metabolomic profiling or fingerprinting of body fluids in livestock animals are based on NMR approaches due to its great robustness and elucidation power (Kullgren et al., 2010; Xu et al., 2015; Jégou et al., 2016; Niu et al., 2016), although one of the main drawbacks of this technique is its low sensitivity (Emwas, 2015). By contrast, MS analyzers coupled to gas chromatography (GC) or high-performance liquid chromatography (HPLC) offer a high sensitivity, becoming a highly feasible and informative technique that has demonstrated its potential in human metabolomic studies (Castro-Puyana & Herrero, 2013; Xu et al., 2009). Besides, both NMR- and MS-based metabolomics rely on wide-untargeted approaches, but MS also allows retrospective analysis of relevant metabolites by means of the full-spectra acquisition by quadrupole time-of-flight mass analyzer (QTOF). Taking in mind all these constraints and advantages, a major aim of this study was to demonstrate the validity of metabolomics based on ultra-high performance liquid chromatography (UHPLC) and high resolution MS (HRMS) to provide new insights on the nutritional and metabolic phenotyping of farmed fish. To this end, the present work was conceived as a MS approach to identify and, most importantly, validate robust biomarkers of malnutrition in short-term fasted fish, using gilthead sea bream (*Sparus aurata*) as a model of a highly cultured fish in all the Mediterranean basin.

## Materials & Methods

### Reagents and chemicals

HPLC-grade water was obtained from a Mili-Q water purification system (Millipore Ltd., Bedford, MA, USA). HPLC-grade methanol (MeOH), HPLC-supergradient acetonitrile (ACN), sodium hydroxide (>99%) and reagent grade ammonium acetate (NH_4_Ac) were obtained from Scharlab (Barcelona, Spain). Leucine-enkephalin (mass-axis calibration), formic acid (mobile phase modifier) and analytical-grade standards methionine sulfoxide and trimethylamine N-oxide were purchased from Sigma-Aldrich (Saint Louis, MO, USA).

### Animal care and sampling

Two-year-old gilthead sea bream of Atlantic origin (average initial weight: 380 g) were reared from early life stages in the indoor experimental facilities of the Institute of Aquaculture Torre de la Sal (IATS), following natural light and temperature conditions at our latitude (40°5′N, 0°10′E). The oxygen content of water was always higher than 85% saturation, unionized ammonia remained below toxic levels (<0.02 mg/l), and rearing density was maintained lower than 15 kg/m^3^.

At mid-summer (July 2014), 30 fish were randomly allocated in two tanks (500 L). One group continued to be fed with a standard commercial diet (Biomar, EFICO Forte 824) to visual satiety one time per day, whereas the other group remained unfed for a 10-day period. At the end of this period, 10 fish from fasted and fed groups (following overnight fasting) were randomly sampled and anaesthetized with 100 mg/L of aminobenzoic acid ethyl ester (MS-222, Sigma-Aldrich) for blood and tissue sampling. Blood was taken from caudal vessels with vacutainer tubes with a clot activator. Liver and visceral adipose tissue were extracted and weighed. Blood samples were allowed to clot for 30 min at room temperature, and then centrifuged at 1,300 g for 10 min. The obtained samples were stored at −20 °C until analysis.

All procedures were approved by the IATS Ethics and Animal Welfare Committee according to national (Royal Decree RD53/2013) and EU legislation (2010/63/EU) on the handling of animals for experiments.

### Sample processing

Serum samples were centrifuged at 12,500 g for 10 min. Supernatant (400 µL) was diluted with 1.2 mL of ACN followed by centrifugation (12,500 g for 10 min). Then, 750 µL of supernatant were stored for hydrophilic interaction liquid chromatography (HILIC), and another 750 µL aliquot was evaporated to dryness by MiVac Duo Concentrator (40 °C, 60 min) and dissolved with MeOH (75 µL) and Mili-Q Water (675 µL) for reversed phase (RP) analysis (details in Fig. S1). Quality control (QC) samples were prepared by pooling 50 µL of each sample extract. All samples were stored at −20 °C until injection.

### UHPLC-HRMS

A Waters Acquity UPLC system (Waters, Milford, MA, USA) was coupled to a hybrid quadrupole-TOF mass spectrometer (Xevo G2 QTOF, Waters, Manchester, UK), using a Z-spray-ESI interface operating in positive and negative ionization mode. The UHPLC separation was performed using Acquity UPLC^®^ BEH C18 1.7 µm particle size analytical column 100 × 2.1 mm (Waters) at 300 µL/min flow rate for RP analysis. An Acquity UPLC^®^ HILIC 1.7 µm particle size analytical column 100 × 2.1 mm (Waters) at 300 µL/min flow rate was used for hydrophilic interaction phase separations.

Each serum sample was injected four times, depending on the procedure (RP and HILIC) and the ionization mode selected (ESI+ and ESI−). The RP separation was performed using H_2_O with 0.01% formic acid (HCOOH) as weak mobile phase (A) and MeOH with 0.01% HCOOH as strong mobile phase (B). The percentage of B was changed from 10% at 0 min, to 90% at 14 min, 90% at 16 min and 10% at 16.01 min, with a total run time of 18 min for both ESI+ and ESI−. For HILIC separation, the weak mobile phase was a mix of ACN:H_2_O (95:5, v/v) with 0.01% HCOOH and 10 mM NH_4_Ac (A), and the strong mobile phase was H_2_O with 0.01% HCOOH and 10 mM NH_4_Ac (B). The B percentage was changed as follows: 0 min, 2%; 1.5 min, 2%; 2.5 min, 15%; 6 min, 50%; 7.5 min, 75%; and finally at 7.51 min, 2%, with a total run time of 10 min, for both ESI+ and ESI−. Sample injection volume was 10 µL in all cases. Nitrogen was used as both the desolvation gas and the nebulizing gas. A capillary voltage of 0.7 kV and 1.5 kV for positive and negative ion modes, respectively, and cone voltage of 25 V were used. MS data were acquired over a *m*∕*z* range of 50–1,200. TOF-MS resolution was approximately 20,000 at full width half maximum at *m*∕*z* 556.2771. Collision gas was argon 99.995% (Praxair, Valencia, Spain). The desolvation gas flow was set at 1,000 L/h, and the cone gas was set at 80 L/h. Desolvation gas temperature was set to 600 °C, source temperature to 130°C and column temperature to 40°C.

For MS^E^ experiments, two acquisition functions with different collision energies were created. The low energy (LE) function, with a fixed collision energy of 4 eV, and the high energy (HE) function, with a collision energy ramp ranging from 15 to 40 eV in order to obtain the (de)protonated ion from LE function and a wide range of fragment ions from the HE function. Both LE and HE functions used a scan time of 0.3 s with an inter-scan delay of 0.05 s. MS/MS experiments were carried out in the same conditions with different collision energies depending on the fragmentation observed for each compound. Calibrations were conducted from *m*∕*z* 50 to 1,200 with a 1:1 mixture of 0.05 M NaOH:5% HCOOH diluted (1:25) with H_2_O:ACN (20:80), at a flow rate of 10  µL/min. For automated accurate mass measurement, a leucine-enkephalin solution (0.5 µg/mL) in ACN:H_2_O (50:50) at 0.1% HCOOH was pumped at 30  µL/min through the lock-spray needle and measured every 30 s, with a scan time of 0.3 s. The (de)protonated molecule of leucine-enkephalin, at *m*∕*z* 556.2771 in positive mode and *m*∕*z* 554.2615 in negative mode was used for recalibrating the mass axis during the injection and to ensure a robust accurate mass along time.

**Figure 1:**
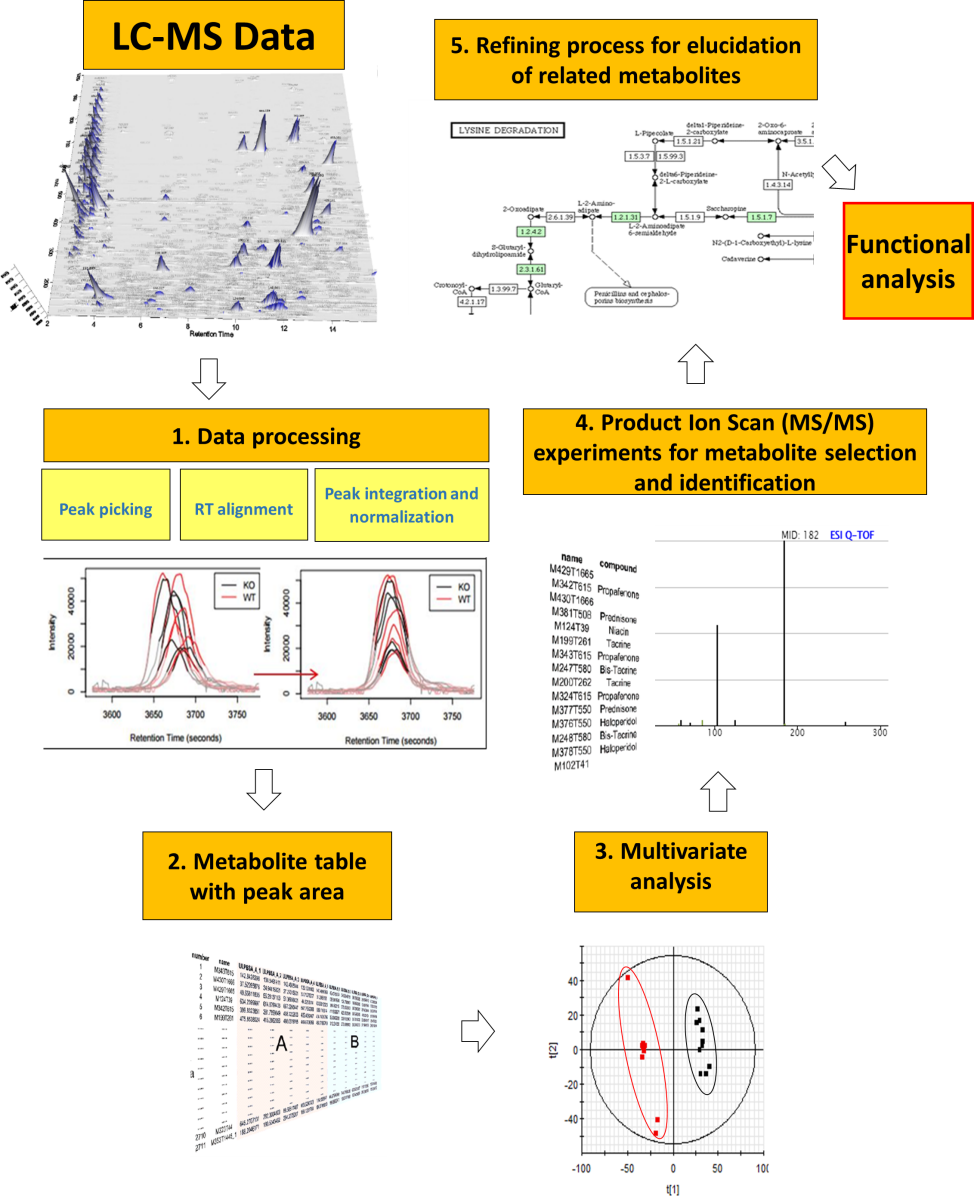
General metabolomics workflow from data acquisition by LC-MS to functional analysis.

### Data processing

The workflow of data processing is shown in Fig. 1. LC-MS spectral data were converted from proprietary (*.raw,* Waters Corp.) to generic *(.cdf,* NetCDF) format using Databridge application (within MassLynx v 4.1; Waters Corporation) and processed using XCMS R package (https://xcmsonline.scripps.edu/) (Smith et al., 2006). *Centwave* feature detection algorithm was employed for peak picking (peak width from 5 to 20 s, S/N ratio higher than 10 and mass tolerance of 15 ppm) followed by retention time alignment for the detected features. Peak area normalization (mean centering) was applied to each data set in order to minimize instrumental drifts with a final log2 transformation to the area to standardize the range of independent feature variance followed by pareto scaling. ANOVA analysis followed by Benjamini–Hochberg multiple testing correction was applied to the normalized peak areas of all metabolites to assess differences between fed and control groups.

Multivariate analysis of processed metabolomics data was performed by means of the EZ-Info software (Umetrics, Sweden). First, Principal Component Analysis (PCA) was employed to ensure the absence of outliers and the correct classification of QCs after normalization. Partial Least Squares—Discriminant analysis (PLS-DA) was then applied to maximize the separation of fed and fasted individuals (Fonville et al., 2010). Orthogonal PLS-DA (OPLS-DA) was also carried out (Wiklund et al., 2008) with a high threshold (*P*[corr] > 0.95) for highlighting the most robust biomarkers.

For elucidation, the MS/MS spectra of the most significant metabolites were compared with reference spectra databases (METLIN, http://metlin.scripps.edu; Human Metabolome DataBase, http://www.hmbd.ca; MassBank, http://www.massbank.eu). For unassigned metabolites, *in silico* fragmentation software (MetFrag, http://msbi.ipb-halle.de/MetFrag) was employed, with subsequent searches through Chemspider (http://www.chemspider.com) and PubChem (https://pubchem.ncbi.nlm.nih.gov) chemical databases. Injection of standards of methionine sulfoxide and trimethylamine N-oxide served to validate the elucidation workflow.

A retrospective analysis of data previously acquired in MS^E^ mode served for the refined search of additional relevant metabolites. It consisted in the search of the *m*∕*z* ratio (parent ions) of the metabolites of interest in the LE function as well as product ions obtained from MS/MS spectrum online databases (METLIN and Human Metabolome DataBase) in the HE function. Integrated areas of each candidate (parent ion) were compared in samples from fed and fasted groups.

## Results & Discussion

### Biometric data

At the end of the experimental period, body weight of fed fish was 15% higher than in fasted fish. This fasting protocol reduced the body fat depots, decreasing significantly (*P* < 0.001) the hepatosomatic index (100 × liver weight/body weight) from 1.3 to 0.9. The similar trend was found for mesenteric fat, although the decrease of mesenteric fat index (100 × mesenteric fat weight/body weight) from 1.9 to 1.6 was not statistically significant (Table 1). The magnitude of these changes was on the range of expected values for one- and two-year-old fish under similar experimental conditions (Benedito-Palos, Ballester-Lozano & Pérez-Sánchez, 2014; Bermejo-Nogales, Calduch-Giner & Pérez-Sánchez, 2015).

**Table 1:** Biometry of fed (control) and fasted gilthead sea bream.

	Control	Fasted	*P*-value
Body weight (g)	426.5 ± 14.1	361.6 ± 10.7	0.002
Length (cm)	24.5 ± 0.3	24.0 ± 0.2	0.196
Condition factor	2.91 ± 0.06	2.63 ± 0.07	0.008
Liver weight (g)	5.63 ± 0.33	3.24 ± 0.16	4E−6
Mesenteric fat (g)	7.20 ± 1.08	6.01 ± 0.89	0.408
HSI (%)^a^	1.32 ± 0.05	0.90 ± 0.03	1E−6
MSI (%)^b^	1.90 ± 0.21	1.64 ± 0.23	0.394

**Notes.**

a Hepatosomatic index = (100 × liver weight)/body weight.

b Mesenteric fat index = (100 × mesenteric fat)/body weight.

### Untargeted metabolomics fingerprinting

Despite of the great potential of GC for chromatographic separation, the nature of serum samples, with medium-high polar compounds in a water-based fluid, pointed out to LC as a more convenient separation technique. UHPLC with sub-2 µm particle size was applied due to its high reproducibility and high separation performance in short-run time analyses. The use of different chromatographic techniques is a key issue to achieve a maximum of detected features when dealing with complex matrices like blood. In our case, serum samples were analyzed with two ionization modes and two different chromatographic columns: RP for a better separation of non-polar compounds, and HILIC to best separate the most polar compounds. In the RP analysis, 6,961 and 3,047 features were detected in both positive and negative ionization modes, while 4,820 and 1,015 features were labeled by XCMS using HILIC separation. This high total number of detected features (*m*∕*z* values) highlights the huge detection power and sensitivity of HRMS and makes feasible a wide-view of sample composition to discriminate the most robust markers of nutritional conditions. Many features were only observed under a single ionization mode and chromatography type, reinforcing the importance of employing different chromatographic columns. As an example, a single peak was detected by HILIC for the significant feature elucidated as LysoPC(20:5) while RP chromatography was able to separate *ω*-3 and *ω*-6 isomers (Fig. S2).

**Figure 2:**
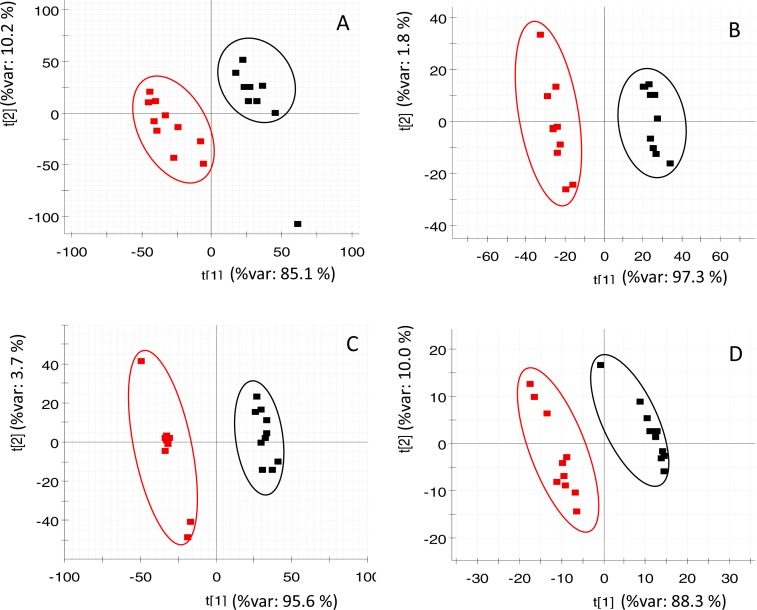
PLS-DA score plots of acquired data of fasted (red) and control (black) fish. *X*-axis corresponds to first component and *Y*-axis to second component (A) RP at positive ionization mode (B) RP at negative ionization mode (C) HILIC at positive ionization mode (D) HILIC in negative ionization mode.

PLS-DA (of RP and HILIC in both positive and negative ionization modes) clearly discriminated the fasted individuals from those of the fed group (Fig. 2). Both groups were separated along the first component of the analysis, which explained 85–97% of the total variance. Individuals of the same group were distributed along the second PLS-DA component (2–10% of total variance). In the case of OPLS-DA, around 850 features from all four datasets were highlighted as discriminatory between fed and fasted fish with a *P*[corr] > 0.95 and a corrected *P*-value < 0.05 (see Fig. S3). Among them, up to 45 different compounds were elucidated as amino acids (4), oligopeptides (8), urea cycle-related metabolites (2), acylcarnitines (5), glutathione-related compounds (5), fatty acids (5), 3-hydroxyisovaleric acid, 3-methoxy-4-hydroxy-phenylglycol (MOPEG) sulphate and phospholipids (14), including phosphatidylcholines (PC) and lysoPC (Table 2).

**Table 2:** Compound list obtained from untargeted approach and refining process.

	Compound name	Biological process^a^	Chromatography/ ionization mode^b^	Formula	De/protonated molecule *m*∕*z* (error mDa)	RT (min)	Change (%) from CTRL^c^	Corrected *P*-value^d^
1	Octanoyl-L-carnitine	1, 2	RP/+	C_15_H_29_NO_4_	288.2158 (−1.8)	10.38	373%	3.1E–06
2	Decanoyl-L-carnitine	1, 2	RP/+	C_17_H_33_NO_4_	316.2478 (−1.0)	12.58	492%	4.53E–07
3	Hexadecenedioic acid mono-L-carnitine ester	1, 2	RP/+	C_23_H_43_NO_6_	430.3157 (−1.2)	13.28	373%	1.15E–06
4	Tetradecadien-L-carnitine	1, 2	RP/+	C_21_H_37_NO_4_	368.2784 (−1.7)	8.90	373%	1.25E–05
5	Tetradecenoyl-L-carnitine	1, 2	RP/+	C_21_H_39_NO_4_	370.2947 (−1.0)	14.88	373%	9.98E–06
6	L-ornithine	3, 4	RP/+	C_5_H_12_N_2_O_2_	133.0972 (−0.5)	5.29	1213%	1.26E–10
7	Citrulline^e^	3, 4	HI/+	C_6_H_13_N_3_O_3_	176.1029 (−0.6)	5.30	140%	5.02E–03
8	Argininosuccinate^e^	3, 4	HI/−	C_10_H_18_N_4_O_6_	289.1145 (−0.3)	1.31	248%	2.07E–02
9	Norvaline	3, 4	RP/−	C_5_H_11_NO_2_	116.0711 (−0.1)	2.51	29%	5.54E–08
10	L- Arginine^e^	3, 4	HI/+	C_6_H_14_N_4_O_2_	175.1194 (−0.1)	6.14	128%	3.37E–02
11	N-(2-cyanoethyl)glycine	3	HI/+	C_5_H_8_N_2_O_2_	129.0673 (+0.9)	5.25	246%	6.05E–09
12	Isoleucine	3	RP/−	C_6_H_13_NO_2_	130.0880 (+1.4)	1.61	230%	1.15E–06
13	Glutamine	3	HI/+	C_5_H_10_N_2_O_3_	147.0766 (−0.4)	5.15	214%	1.80E–05
14	Glu-Phe	3	RP/+	C_14_H_18_N_2_O_5_	295.1278 (−1.6)	4.61	23%	9.11E–07
15	His-Phe	3	RP/+	C_15_H_18_N_4_O_3_	303.1447 (−1.0)	2.08	50%	6.53E–05
16	LTYV	3	RP/−	C_24_H_38_N_4_O_7_	493.2652 (−1.0)	5.80	23%	9.98E–06
17	LLGGPS	3	RP/+	C_24_H_42_N_6_O_8_	543.3148 (+0.6)	6.48	214%	2.53E–05
18	QLWD	3	RP/+	C_26_H_36_N_6_O_8_	561.2688 (+1.5)	8.08	373%	4.58E–04
19	YLWV	3	RP/+	C_24_H_38_N_4_O_7_	495.2807 (−1.2)	8.10	283%	2.77E–04
20	SVLGPA	3	RP/+	C_24_H_42_N_6_O_8_	543.3141 (−0.1)	14.75	746%	2.87E–07
21	N(2-Furoyl)glycyl-leucine	3	RP/−	C_13_H_20_N_2_O_5_	283.1301 (+0.7)	5.15	303%	3.44E–09
22	MOPEG sulphate	5	RP/−	C_9_H_12_O_7_S	263.0223 (−0.2)	2.01	325%	9.70E–07
23	Noradrenaline^e^	5	HI/+	C_8_H_11_NO_3_	170.0806 (−1.1)	3.02	141%	9.34E–03
24	DOPEGAL^e^	5	HI/+	C_8_H_8_O_4_	169.0501 (0.0)	3.11	229%	3.94E–07
25	DOPEG^e^	5	HI/+	C_8_H_10_O_4_	171.0649 (−0.8)	2.55	108%	1.94E–01
26	ϒ-Glu-Leu	6	HI/+	C_11_H_20_N_2_O_5_	261.1432 (−1.8)	3.37	246%	3.13E–06
27	ϒ-Glu-Val	6	RP/+	C_10_H_18_N_2_O_5_	247.1285 (−0.9)	2.15	246%	1.05E–08
28	ϒ-Glu-Ile	6	HI/+	C_11_H_20_N_2_O_5_	261.1446 (−0.4)	3.12	696%	1.48E–08
29	Pyroglutamic acid	6	HI/+	C_5_H_7_NO_3_	130.0521 (−1.7)	5.15	303%	4.26E–06
30	Glutathione^e^	6	HI/−	C_10_H_17_N_3_O_6_S	306.0760 (0.0)	1.37	66%	3.03E–02
31	*γ*-Glu-Cys^e^	6	HI/+	C_8_H_14_N_2_O_5_S	251.0701 (−0.1)	4.92	63%	4.22E–01
32	Methionine sulfoxide	6	HI/+	C_5_H_11_NO_3_S	166.0511 (−0.2)	4.96	41%	2.08E–04
33	FFA (C18:3)	1, 2	RP/−	C_18_H_30_O_2_	277.2172 (−0.4)	15.41	246%	2.08E–04
34	9-hydroxy-octadecanoic acid	1, 2	RP/−	C_18_H_36_O_3_	299.2598 (+1.3)	14.98	528%	2.23E–07
35	Linoleic acid	1, 2	RP/−	C_20_H_34_O_2_	305.2500 (+1.9)	16.31	19%	3.43E–06
36	Eicosapentaenoic acid	1, 2	RP/−	C_20_H_30_O_2_	301.2152 (−1.6)	15.10	33%	2.01E–03
37	Acetohexadecyloxy propylaminoethyl phosphate	1, 7	RP/+	C_23_H_48_NO_7_P	482.3250 (+0.3)	14.93	35%	2.53E–06
38	LysoPC(14:0)	1, 7	RP/+	C_22_H_46_NO_7_P	468.3083 (−0.7)	15.41	25%	9.29E–05
39	LysoPC(16:1)	1, 7	RP/+	C_24_H_48_NO_7_P	494.3244 (−0.3)	14.91	44%	1.71E–04
40	LysoPC(18:4)	1, 7	RP/+	C_26_H_46_NO_7_P	516.3079 (−1.1)	14.36	11%	1.40E–08
41	LysoPC(18:3)	1, 7	RP/+	C_26_H_48_NO_7_P	518.3247 (0.0)	14.38	4%	1.93E–11
42	LysoPC(20:5)	1, 7	RP/+	C_28_H_48_NO_7_P	542.3248 (+0.1)	14.58	13%	1.37E–08
43	LysoPC(18:1)	1, 7	RP/+	C_26_H_52_NO_7_P	522.3560 (0.0)	15.00	16%	8.10E–08
44	LysoPC(21:5)	1, 7	RP/+	C_29_H_50_NO_7_P	556.3408 (+0.5)	14.88	31%	9.98E–06
45	LysoPC(20:4)	1, 7	RP/+	C_28_H_50_NO_7_P	544.3411 (+0.8)	14.06	20%	7.01E–06
46	LysoPC (Tetradecylthioacetic acid)	1, 7	RP/+	C_24_H_48_NO_7_PS	526.2951 (−1.6)	15.13	1%	1.62E–09
47	PC(22:5/20:5)	1, 7	HI/+	C_50_H_80_NO_8_P	854.5689 (−1.1)	4.11	27%	3.01E–08
48	PC(20:5/18:1)	1, 7	HI/+	C_46_H_78_NO_8_P	804.5435 (−0.8)	6.78	200%	1.95E–02
49	PC(20:4/18:1)	1, 7	HI/+	C_46_H_82_NO_8_P	808.5843 (−1.3)	5.73	214%	4.69E–02
50	PC(18:1/16:0)	1, 7	HI/+	C_42_H_82_NO_8_P	760.5847 (−0.9)	4.06	303%	1.79E–05
51	TMAO	1, 7	HI/+	C_3_H_9_NO	76.0760 (−0.2)	5.85	41%	7.68E–05
52	Glycerophos phocholine	1, 7	HI/+	C_8_H_20_NO_6_P	258.1106 (0.0)	5.73	12%	5.69E–05
53	Hydroxyisovaleric acid	8	RP/−	C_5_H_10_O_3_	117.0553 (+0.1)	2.85	373%	2.41E–09
54	Biotin^e^	8	RP/+	C_10_H_16_N_2_O_3_S	245.0959 (−0.1)	5.40	80%	2.46E–01

**Notes.**

a 1, Lipid metabolism; 2, Fatty acid metabolism; 3, Protein metabolism; 4, Amino catabolism/urea-cycle; 5, Stress response/catecholamine metabolism; 6, Oxidative stress/glutathione metabolism; 7, Phospholipid metabolism; 8, Vitamin metabolism.

b Chromatography and ionization modes in which the signal area was higher for the highlighted compound.

c Variation of area between fed and fasted fish. Variation > 100% means higher area in fasted fish, and <100% means lower are in fasted fish.

d ANOVA followed by Benjamini–Hochberg multiple testing correction.

e Compounds obtained in refining process.

Phospholipids were characterized by the presence of both the protonated molecule and sodium adduct in the positive LE spectra and their acetate adducts in negative LE spectra. As an example, Fig. S4 shows the *m*∕*z* 542.3248 (+0.1 mDa mass error) which corresponds to the protonated molecule, and *m*∕*z* 564.3074 (+1.2 mDa mass error) corresponds to the sodium adduct in RP+. MS/MS experiments were also carried out obtaining *m*∕*z*184.0735 (−0.4 mDa) (main product ions at 30 eV) in positive ionization mode. These *m*∕*z* ions were annotated as phosphocholine in agreement with fragmentation pathways of lysoPC (Xu et al., 2009; González-Domínguez, García-Barrera & Gómez-Ariza, 2014).

Carnitine-related compounds were also elucidated after MS/MS experiments by the presence of *m*∕*z* 144.1051 (C_7_H_13_NO${}_{2}^{\mathrm{+}}$, +2.6 mDa), 85.0303 (C_4_H_5_O${}_{2}^{\mathrm{+}}$, +1.3 mDa) and 60.0814 (C_3_H_10_N^+^, −0.3 mDa) as characteristic product ions of these compounds (Möder et al., 1997; Luci, Hirche & Eder, 2008) also observed in METLIN spectra for some of these compounds (Fig. S5).

Tandem mass spectrometry also provides relevant information for isomers differentiation. For example, *γ*-Glu-Ile and *γ*-Glu-Leu presented the same molecular formula and close retention times (Fig. S6). MS/MS experiments revealed very similar spectra at 10 and 20 eV with the exception of the *m*∕*z* 142.0499 which only appeared for the peak at 3.37 min. After comparing both spectra with METLIN, only *γ*-Glu-Leu spectrum showed this *m*∕*z* ion at 20 eV. The formation of C_4_H${}_{8}^{+}$ have been observed and described in the literature much higher in isoleucine than in leucine (Squire, Beranová & Wesdemiotis, 1995), so in leucine spectra the neutral loss of C_4_H_8_ can be observed while it does not appears in isoleucine spectra. This strategy was followed for the rest of elucidated compounds.

### Functional analysis of elucidated compounds

Biological significance of the concurrent up- or down-regulation of most of the elucidated metabolites during fasting clearly stated that food deprivation increased mobilization of body energy stores and improved the oxidative capacity of metabolic fuels, which paralleled the onset of specific changes in the cell redox-balance. In this regard, the increased mobilization of body fat stores in fasted individuals, exemplified by the loss of liver and adipose tissue mass, was linked to the consistent increase of circulating levels of five sub-products of L-carnitine (compounds 1–5 in Table 2), a carrier of fatty acids across the inner mitochondrial membrane for their subsequent beta-oxidation (Luci, Hirche & Eder, 2008; Ball, Urschel & Pencharz, 2007). At the molecular level, this was early substantiated in similar experimental conditions by a marked up-regulated expression of the two carnitine palmitoyltransferase variants (CPT1A, CPT1B) of the skeletal muscle of gilthead sea bream (Benedito-Palos, Ballester-Lozano & Pérez-Sánchez, 2014), which was encompassed by the increased expression of a high representation (25 enzyme subunits) of regulatory and assembly factors of the five enzyme complex units (Complex I–V) of the mitochondrial respiratory chain (Bermejo-Nogales, Calduch-Giner & Pérez-Sánchez, 2015). Microarray gene expression profiling of either glycolytic or aerobic muscle tissues of fish fed to maintenance ration also indicates that nutrient scarcity is by itself a major factor driving switches in muscle protein turnover and mitochondrial activity (Calduch-Giner et al., 2014). In the present study, this was reinforced by the consistent fasting increase of serum concentrations of urea cycle-related compounds (citrulline, ornithine, argininosuccinate and arginine). Of note, the activity of urea cycle enzymes is typically higher in carnivorous fish than in herbivorous and omnivorous fish species (Chiu, Austic & Rumsey, 1986), and our results highlighted that acyl-carnitine and urea cycle metabolites are specially sensitive to fasting-mediated changes in fatty acid and amino acid catabolism during negative energy balance.

Catecholamines are mobilized into fish circulation during a variety of stressful situations which require modulation of cardiorespiratory function or mobilization of energy reserves. The magnitude of change is dependent on the species and the type and intensity of stress imposed, although a wide range of stressors including hypoxia, hypercapnia, exhaustive and violent exercise, air exposure or anemia are considered strong activators of the hypothalamic-pituitary-interrenal (HPI) axis in fish (Reid, Bernier & Perry, 1998). This also applies to short-term fasting (De Pedro et al., 2003), and the observed increase of MOPEG sulphate, a metabolite of norepinephrine degradation, can be viewed as part of the adaptive response of the HPI axis to cope with fasting hypoglycemia through the activation of lipolysis and gluconeogenesis. This notion was supported by the increased levels of noradrenaline, and other catecholamine metabolites 3,4-dihydroxymandelaldehyde and 3,4-dihydroxyphenylethyleneglycol (DOPEGAL and DOPEG), detected in the refined search step of analysis.

**Figure 3:**
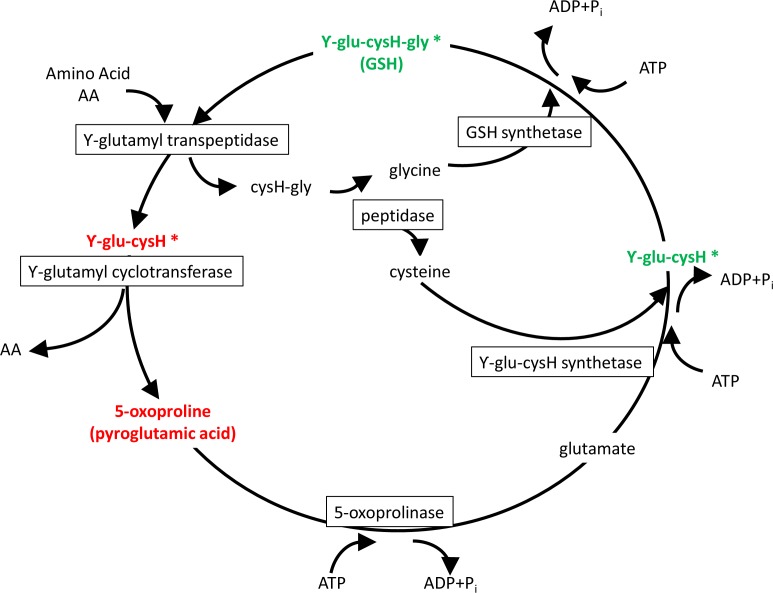
Meister’s cycle. In red, elucidated metabolites up-regulated with fasting; in green, down-regulated with fasting. Asterisks mark elucidated metabolites by means of the refining step.

The primary enzymatic antioxidant defense system of living organisms is the glutathione (GSH) redox system that reduces hydrogen peroxide and lipid hydroperoxides at the expense of oxidizing GSH to its disulfide form (GSSG). Once oxidized, GSH can be reduced back by glutathione reductase, using NADPH as an electron donor, and previous studies in gilthead seam bream indicate that either absolute GSH levels or the GSH/GSSG ratio are regulated by dietary oils, increasing the total plasma antioxidant capacity with the increased unsaturation index of dietary oils of marine origin (Saera-Vila et al., 2005). Likewise, total plasma antioxidant capacity is increased in hypoxic fish with a switch from oxidative phosphorylation (OXPHOS) to anaerobic glycolysis (Bermejo-Nogales, Calduch-Giner & Pérez-Sánchez, 2014), which results in reduced mitochondria oxygen consumption and enhanced NADH production from glycolysis (Frezza et al., 2011). Importantly, extension of life span is related in mammals and birds to low antioxidant levels and low rates of generation of reactive oxygen species (ROS) (Lykkesfeldt & Svendsen, 2007; Pamplona et al., 2008). Experimental evidence in rats also indicates that intermittent fasting affects redox balance in a tissue selective manner (Chausse et al., 2015), and our fish metabolomic study highlighted that the depletion of serum GSH during short-term fasting was closely related to changes in the Meister’s *γ*-glutamyl cycle with a key role in the recovery and delivery of cysteine in the body (Griffith, Bridges & Meister, 1978). This was supported by high circulating concentrations of ϒ-Glu-(Leu/Val/Ile) and pyroglutamic acid in the serum of fasted gilthead sea bream, whereas both GSH and ϒ-Glu-Cys were depleted (Fig. 3). This represents a complex trade-off with a reduced risk of oxidative stress, also highlighted by the decreased concentration of methionine sulfoxide, an oxidized form of methionine that is highly correlated with the risk of oxidative stress (Weissbach et al., 2002). In parallel, other short oligopeptides were either increased or decreased in the serum of fasted fish. It remains to be established if they have a physiological significance or are subproducts of protein hydrolysis.

The fatty acid composition of triacylglycerols (TAG) usually clears a close resemblance to dietary lipids (Benedito-Palos et al., 2010), whereas that of phospholipids is highly regulated and influenced in fish by environmental factors, including temperature and osmolarity (Los & Murata, 2004; Ibarz et al., 2005). The specific effects of ration size have been addressed in gilthead sea bream, and maintenance ratio significantly increased the retention of arachidonic acid (ARA) and docosahexanoic acid (DHA) in muscle phospholipids, whereas the fatty acid composition of storage lipids remained almost unchanged (Benedito-Palos et al., 2013). This lean muscle phenotype was linked in the present study to low plasma levels of linoleic acid and eicosapentaenoic acid, the precursors of ARA and DHA, respectively. At the same time, fasting induced an overall decrease of circulating lysoPC and glycerophosphocholine, whereas the effect on phosphatydylcholines was more selective depending of the composition of fatty acids. In any case, phospholipid metabolism is becoming highly regulated by feed intake at either blood or tissue level. Thus, phospholipids of skeletal muscle would act as a reservoir of long chain poly-unsaturated fatty acids with an enhanced expression of lipoprotein lipase-like, a TAG lipase isoform exclusive of fish linage which is highly expressed in muscle tissues and specifically up-regulated by feed restriction (Benedito-Palos et al., 2013; Rimoldi et al., 2015). This in turn would mediate, at least in part, the changes in the blood composition of phospholipid-related metabolites. This is extensive to trimethylamine N-oxide (TMAO), and high TMAO and choline concentrations are associated in humans with diabetes and advanced cardio-metabolic risk profile (Obeid et al., 2016). In agreement with this, the opposite pattern was found herein in fish under a negative energy balance, which reinforces the close metabolic association between interrelated pathways of phospholipid and oxidative metabolism. Recent metabolomics studies have highlighted a concurrent depletion of reduced GSH and glycerophosphocholine in the gills of another fish species, the golden grey mullet (*Liza aurata*), as a response to mercury toxicity (Cappello et al., 2016a; Cappello et al., 2016b). This finding also reinforces the view that the response to different challenges such as malnutrition or pollutants toxicity and susceptibility (Kokushi et al., 2016; Wang et al., 2016) can be assessed by the analysis of common responsive metabolites, opening the possibility for future screening of the fish general welfare status through selected biomarkers.

**Figure 4:**
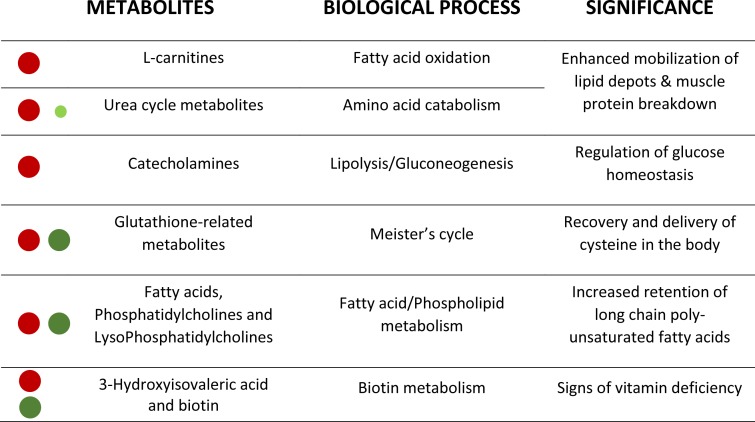
Corollary with the metabolic significance of highlighted metabolites. Red and green circles signals the degree of up- and down-regulation of metabolites, respectively, with fasting.

Lastly, major changes in vitamin status are related in our experimental model to biotin metabolism. In humans, the impairment of renal reclamation of biotin results in an elevated urine concentration of 3-hydroxyisovaleric acid (Mock et al., 1997). Accordingly, we found that the fasting increase of this metabolite was concurrent with a low biotin availability (highlighted by refining analysis), which reinforces the role of 3-hydroxyisovaleric acid as a biomarker of B7 vitamin deficiency in a wide range of vertebrate species, including fish.

## Conclusions

This metabolomics study has been performed to help fish physiologists and nutritionists to identify highly sensitive and robust biomarkers of malnutrition from a large list of affected compounds (about 850 ions) (see Fig. 4 as a corollary). The MS^E^ acquisition mode of the involved QTOF allowed to simultaneously recording both low and high collision energy mass spectra. In this sense, as full scan data is acquired, the possibility of refining steps after elucidation gives HRMS a strong advantage compared to NMR. Further studies are underway to determine the potential of this powerful methodological approach, alone or in combination with other omics approaches, for the discovery and validation of new biomarkers of nutritional status in a wide-range of physiological conditions arising with the advent of new fish feed formulations.

##  Supplemental Information

10.7717/peerj.2920/supp-1 Supplemental Information 1 Supplemental material

10.7717/peerj.2920/supp-2 Supplemental Information 2 Raw data

## Additional Information and Declarations

### Data Availability

The following information was supplied regarding data availability: The raw data has been supplied as Data S1.
