# Supplementary material for: Untargeted metabolomics approach for unraveling robust biomarkers of nutritional status in fasted gilthead sea bream (Sparus aurata)
Source: PeerJ. 2017 Jan 26;5:e2920. doi: 10.7717/peerj.2920 (PMC5291114; doi:10.7717/peerj.2920)
Supplement: Supplemental Information 1 [file peerj-05-2920-s001.txt]

SUPLEMENTARY MATERIAL

**Figure S1**. Sample treatment for fish serum in both reversed phase and hydrophilic interaction liquid chromatography.

12,500 g

**Figure S2:** A extracted ion chromatogram (XIC) for LysoPC(20:5) in HILIC and RP in both positive and negative ionization modes. In RP chromatography the separation of ω-3 and ω-6 LysoPC can be appreciated in both positive and negative ionization mode.


**Figure S3**: Orthogonal PLS-DA S-Plot for individual biomarker highlighting. In red, ions with more than 0.95 correlation between groups. The ions enhanced by fasting are top-right and these ones decreased by fasting bottom-left on the plots. Top-left plot is the RP at positive ionization mode, top-right HILIC at positive ionization mode, bottom-left for RP at negative ionization mode and bottom-right HILIC in negative ionization mode.

**Figure S4**: Chromatogram (A) and mass spectra (B) for LysoPC(20:5) in both low (bottom) and high (top) energy functions in MS^E^ acquisition mode. Protonated and sodium-adduct molecule appears in the LE function (B at bottom) and LysoPC are observed in HE function (B at top)

**Figure S5:** **A:** MS/MS spectra at 10 eV and 20 eV collision energy of compound 2 (in table 3). Ions marked in red are specific for carnitine related compounds. **B:** METLIN spectra for Decanoyl-L-carnitine

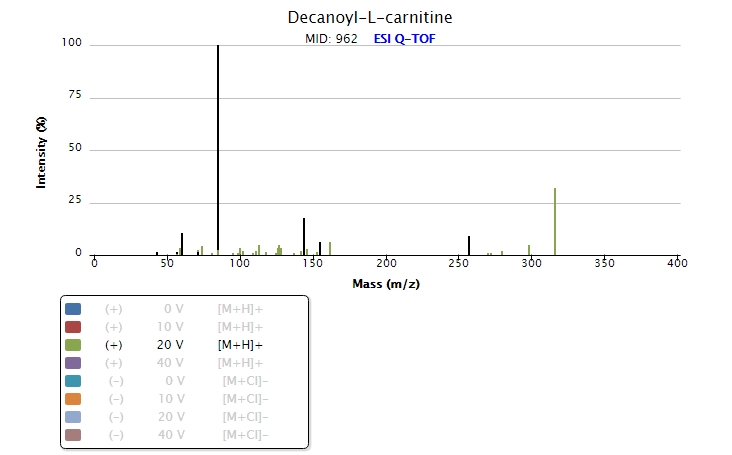


85.0290

60.0817

144.1020

155.1430

257.1747

316.2486

B

A

**Figure S6**: γ-Glu-Ile and γ-Glu-Leu fragmentation patterns which were employed to assign both isomers. **A1**: XIC at m/z 261.143 for a MS^E^ injection, **A2:** XIC from a MS/MS experiment at 20 eV for a common fragment ion of both compounds (γ-Glu-Ile and γ-Glu-Leu). **A3:** XIC from a MS/MS experiment at 20 eV for a fragment ion only occurred in γ -Glu-leu. **B:** MS/MS spectrum isolating m/z 261.1 at 10 eV for γ-Glu-Ile (**B1**), γ-Glu-Leu **(B2**) and at 20 eV for γ-Glu-Leu (**B3**), γ-Glu-Ile (**B4**) which shows the specific fragment ions (**B3**). **C:** METLIN MS/MS spectrum for γ-Glu-Leu. **D:** METLIN MS/MS spectrum for γ-Glu-Ile.

A3

B1

B2

B4

B3

A2

A1

C


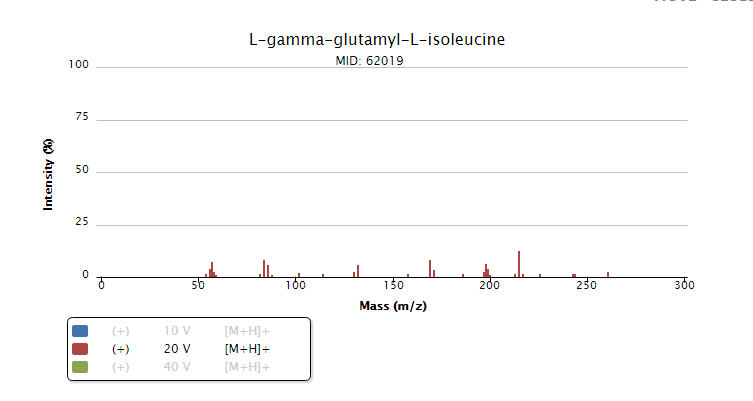

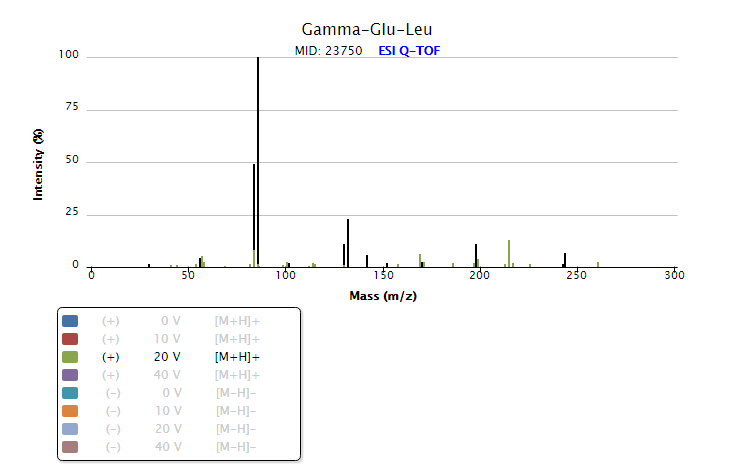


142,0492

142,0492

C

D
